# Spatial dynamics and socioeconomic factors correlated with American cutaneous leishmaniasis in Pernambuco, Brazil from 2008 to 2017

**DOI:** 10.1590/0037-8682-0373-2019

**Published:** 2020-04-27

**Authors:** Andréa Flávia Luckwü de Santana Gonçalves, Suzanne Santos de Lima, Amanda Priscila de Santana Cabral Silva, Celivane Cavalcanti Barbosa

**Affiliations:** 1 Secretaria de Saúde do Recife, Programa de Residência Multiprofissional em Saúde Coletiva, Recife, PE, Brasil.; 2 Fundação Oswaldo Cruz, Instituto Aggeu Magalhães, Departamento de Saúde Coletiva, Recife, PE, Brasil.; 3 Universidade Federal de Pernambuco, Centro Acadêmico de Vitória, Núcleo de Saúde Coletiva, Vitória de Santo Antão, PE, Brasil.; 4 Secretaria de Saúde de Pernambuco, Departamento de Vigilância Epidemiológica da I Regional de Saúde, Recife, PE, Brasil.

**Keywords:** Neglected diseases, Cutaneous leishmaniasis, Public health surveillance, Spatial analysis

## Abstract

**INTRODUCTION:**

American cutaneous leishmaniasis (ACL) is a public health problem and has been associated with country’s territory. We aimed to analyze the spatial dynamics and socioeconomic factors correlated to the incidence of ACL in Pernambuco, Brazil from 2008 to 2017.

**METHODS:**

A cross-sectional, ecological study was conducted in the Brazilian municipalities. Patient data were obtained from the Health Hazard Notification System (SINAN); indicators and incidence for the total period and for quinquennium were obtained. Socioeconomic factors were analyzed to evaluate the association between the incidence of ACL and presence of bathroom and running water, garbage collection availability, inadequate water supply, sanitation, rural population, per capita income, and vulnerability to poverty. Spatial analysis considered the gross incidence; the Bayesian local empirical method and Moran spatial autocorrelation index were applied using Terra View and QGIS.

**RESULTS:**

The incidence of ACL reduced (0.29/100,000 inhabitants per year). Individuals with ACL were young adults (30.3%), men (60.2%), brown skinned (62.9%), rural residents (70.6%), and less educated (46.7%); had autochthonous transmission (78.8%); developed the cutaneous form (97.2%); had evolution to cure (82.7%); and were diagnosed using the clinical epidemiological criterion (70.5%). ACL occurred in the large part of the state and showed heterogeneous distribution, with persistence of two high priority intervention clusters covering Health Regions I, II, III, IV, and XII.

**CONCLUSIONS:**

Spatial analysis and epidemiological indicators complement each other. The combination of these methods can improve the understanding on ACL occurrence, which will help subsidize planning and enhance the quality and effectiveness of healthcare interventions.

## INTRODUCTION

Leishmaniasis is a relevant public health issue due to its epidemiological diversity, high incidence, transcendence, and wide geographical distribution; it is one of the most frequent neglected parasitic infections in the world^1^ ^,^ ^2^. The disease has two clinical forms-visceral leishmaniasis (VL) and American cutaneous leishmaniasis (ACL)^3^.

ACL is widely distributed worldwide, and it is endemic in 87 countries and territories. More than two-thirds of new cases of the patients live in the following nine countries, mainly affecting the socioeconomically disadvantaged people^5^: Afghanistan, Algeria, Brazil, Colombia, Islamic Republic of Iran, Pakistan, Peru, Saudi Arabia, and Syrian Arab Republic^4^. In the Americas, 73% of the cases were registered in four countries^6^: Brazil (35.1%) followed by Colombia (15.5%), Peru (13.3%), and Nicaragua (8.7%).

Brazil had an extremely high ACL transmission rate based on the Leishmaniasis Composite Index (17.7/100,000 inhabitants); this index considers the incidence and density of the cases from 2015 to 2017 triennium^6^. The disease is widespread in all Brazilian states and presents various epidemiological profiles and transmission patterns associated with socioenvironmental modifications^7^. Approximately 25% of the country’s cases were reported in the Brazilian Northeast, with the highest incidence in Bahia, Maranhão, Ceará, and Pernambuco^3^.

Efforts have been made to reduce the occurrence of ACL. In 2017, the Leishmaniasis Plan of Action for the Americas 2017-2022 was undertaken to reduce morbidity and mortality by strengthening diagnosis, treatment, rehabilitation, prevention, surveillance, and control^8^. In Brazil, the National Surveillance Program for Cutaneous Leishmaniasis was instituted to reduce morbidity, deformities, and deaths^3^. In addition, is a Compulsory Notification Disease; therefore, a structured surveillance is implemented, and its notification makes it possible to measure the magnitude, distribution, and behavior of the disease^3^.

It is worth highlighting that ACL occurs through a close interaction between physical-geographical, biological (vector, host, and parasite), and socioeconomic factors^9^. The first deals with the relationship among health, society, and environment; it is influenced by anthropic actions associated with deforestation and accelerated urbanization process^10^ ^,^ ^11^. Favored by such conditions, biological factors are responsible for the vector adaptation at the reservoirs and environments; in these spots, dispersion, invasion, and adaptation of the vector to urban/peri-urban areas has been facilitated^12^ ^,^ ^13^.

The population’s socioeconomic conditions can increase the disease burden and prolong its clinical course^14^ ^-^ ^17^. Studies bespeak that complex dimension of poverty has been an important risk factor for the ACL occurrence-social inequalities and vulnerability, Human Development Index, occupation, and education^12^ ^,^ ^13^ ^,^ ^18^ ^,^ ^19^. Besides, ACL social determinants are closely linked to spatial dynamics such as the area characteristics and the geographical aggregation patterns^11^.

ACL occurrence has been closely related to territory and its complex characteristics. However, as data on this subject are limited, further studies are warranted. Hence, this study aimed to analyze the spatial dynamic and socioeconomic factors associated with ACL incidence in the state of Pernambuco, from 2008 to 2017.

## METHODS

### Design and study area

We conducted a mixed descriptive study, cross-sectional and ecological type. The study aimed to describe the sociodemographic and clinical profile as well as to verify the correlations between exposure and the occurrence of disease in a population belonging to a defined geographical area.

The study was conducted in Pernambuco, located in Brazilian Northeast region, with an area of 98.076.001km^2^ and an estimated population of 9.473.266 inhabitants^20^. The state is composed of 184 municipalities and a state district (Fernando de Noronha); it is grouped under the logic of regionalization in 12 Health Regions^21^. 

In this administrative division, which is heterogeneous, municipalities with similar cultural, sociodemographic, environmental, and economic characteristics are grouped together. The Health Regions II, III, and XII make up Zona da Mata; IV, V, and VI make up Agreste; and VII, IX, X, and XI make up Pernambuco’s Sertão. Meanwhile, Health Region VIII is in Pernambuco’s São Francisco. 

### Data management and analysis

The secondary data recorded in the Health Hazard Notification System (*Sistema de Informação de Notificação de Agravos de Notificação Compulsória [SINAN]*) were extracted from the compulsory notification report of ACL; the report is a model standardized by the Ministry of Health. The cases that were selected in this study were confirmed based on the entry types: “new case” and “resident in Pernambuco state” diagnosed between January 1, 2008 and December 31, 2017. A total of 3,943 cases were included in the study. After analyzing the inconsistencies and duplications using the Statistical Package for the Social Sciences version 21.0, 449 (11,4%) cases were excluded from the study.

Population data and cartographic meshes were obtained from the Brazilian Institute of Geography and Statistics (IBGE). Data on socioeconomic indicators were obtained from the Brazilian Human Development Atlas of the United Nations Development Program and the Database of Pernambuco State based on the year of the last census, 2010.

The variables to delineate sociodemographic profile and epidemiological and clinical characteristics profile of ACL patients were analyzed according to completeness in accordance with the criteria provided by Oliveira et al. (2009)^22^. As stated in the postulate, “occupancy” and “HIV-coinfection” were excluded from the analysis as they presented low completeness (<70%). Then, absolute and relative frequency rates were calculated. 

The incidence rates of ACL in the municipality and in Pernambuco state within the entire time interval (2008-2017) and within the two quinquennium aggregations (2008-2012 and 2013-2017) were determined. Variations in the incidence percentage were calculated considering the extreme years. The trend of the incidence was verified using Prais-Winsten temporal regression^23^. 

To explore the socioeconomic factors and ACL, the zero-adjusted gamma regression model (ZAGA) was used. This model was chosen because the response variable has a relevant number of zero (about 68.5%) and because it is strictly positive. The incidence of ACL in 2010 was evaluated because the explanatory variables were obtained from the 2010 IBGE census^20^, which mitigates the possible temporal correlation problem.

The adjusted range model of zeros has three parameters-µ (location parameter or mean), σ (variance component), and ν (probability that the modeled variable will obtain values equal to zero). The connection functions for each of the abovementioned parameter are log, log, and logit, respectively^24^.

The socioeconomic variables used were: Percentage of the population with bathroom and running water (bathroom and water), percentage of people in households with inadequate water supply and sanitation (water and sewage), percentage of the population living in the rural area (pop rural), per capita income (income), and percentage of vulnerable to poverty (vulnerable). 

Some of these variables have a high correlation; because of this, they were subjected to the dimensionality reduction technique using main components^25^. Through the linear combination of the variables that went through this process, a new explanatory variable was created that was normalized for the interval 0-1 and presented as follows, facilitating the interpretation of these new variables:

$$X'=\frac{X-mín(X)}{máx(X)-mín(X)}$$

To form new variables that are more useful for modeling and that can be easily interpreted, two new variables were generated using the equations below:

$$Infrastructure=0,61\left(Bathroom\;and\;water\right)-0,56\left(water\;and\;sewage\right)$$

-0,55(Pop.rural);

$$Poverty=0,71\left(Vulnerable\right)-0,71\left(Income\right)$$

The proportions of variance estimated by the component that generates the variables infrastructure and poverty are 79.9% and 93.1%, respectively.

The infrastructure variable is related to the bathroom and water variables: water and sewage and rural population. It shows the level of infrastructure of municipalities; the higher the percentage of households with piped water and toilets, the greater the value of this variable. As a reflection of the previous fact, the higher the percentage of households in rural areas with inadequate sewage and water supply, the lower the value for this variable. Meanwhile, the new poverty variable measures the level of poverty in the municipalities. That is, the greater the percentage of vulnerable to poverty, the greater its value. Moreover, the greater its value for per capita income, the lower its value.

The software used was R version 3.6.1., GAMLSS packages version 5.1.5 and Prais 1.1.1 for the time trend model.

For spatial analysis, the District of Fernando de Noronha was withdrew from the maps composition because in addition it not present cases in the period; it is an island that do not have bordering municipalities. The gross incidence rates were presented using thematic maps. Seeking to understand the effects of neighboring municipalities on the occurrence of the disease, rates were smoothed using the local empirical Bayesian method, which estimates the coefficients considering the territory of the observed population and the neighboring municipalities.

To verify the existence of spatial dependence, the Global Moran’s Spatial Autocorrelation Index was calculated, and the Spatial Autocorrelation Index Moran Place was analyzed, which included three steps: (1) identification of priority areas, using the Moran scattering diagram to compare the spatial dependence of each municipality, where quadrants are generated with the following interpretation-Q1 (high/high) and Q2 (low/low), which indicate positive or similar spatial association points with its neighbors, and Q3 (high/low) and Q4 (low/high), which indicate negative spatial association points, municipalities that have different values from their neighbors^26^ ^,^ ^27^. This step is visually represented by a BoxMap. 

In the second stage, we used the Local Indicators of Spatial Association (LISA) Indicator, which is used to detect regions with local correlation significantly different from the rest of the data, applying the local spatial autocorrelation statistics. The significance assessment is performed by comparing a series of values ​​obtained by permutating the values of neighboring areas^28^.

The third step mixes the zones that have positive spatial relationship and are identified by the BoxMap (with spatial significance above 95%) and those with positive spatial relationship identified by LisaMap. The combination of these two groups generated the MoranMap. Priority areas were those made up of MoranMap class Q1, classes Q3 and Q4 are transition areas, where they could evolve into areas of high priority intervention if effective action is not taken.

Maps were elaborated using Terra View 4.2.2 and QGIS 2.18.14 programs.

### Ethics considerations

The research project was approved by the Ethics Committee of the Otávio de Freitas Hospital (opinion number: 2.949.196/2018[CAAE: 91560318.5.0000.5200]).

## RESULTS

### Diagnosed cases profile

From 2008 to 2017, 3,493 new ACL cases were reported in the state of Pernambuco. The most significant incidence rate was in 2009 (5,82/100,000 inhabitants) followed by 2015 (4,9/100,000 inhabitants). Although the incidence peaked in 2015, the incidence decreased to 34,2% compared with that in the extreme years of the historical series. When analyzing the two quinquennium groups, a 26.6% reduction was observed in 2008-2012 and a 33,7% reduction was observed in 2013-2017. Prais-Winsten regression showed that the incidence rate reduced to 0,29/100,000 inhabitants (p = 0,02). 

By stratifying the incidence rates of municipalities according to Health Region, we found that Region III had the highest incidence. The incidence rates in Regions V, VIII, XI, and XII Health increased compared with those during two 5-year periods, which is different from the results of the regression analysis, even subtle (Table 1).

With regard to the sociodemographic data, individuals affected by the ACL were aged 20 and 39 years (1,060; 30.3%), were men (2,102; 60.2%), had brown race/color (2,198; 62.9%), had less than elementary education (1,630; 46.7%), and were rural residents (2,467; 70.6%) (Table 2). Epidemiological clinical variables showed that the cutaneous form of the disease was more common among those infected (3,395; 97.2%), with autochthonous cases (2,754; 78.8%), epidemiological clinical criterion confirmation (2,464; 70.5%), and evolution to cure (2,887; 82.7%) (Table 2).

**TABLE 1:** Distribution of annual mean cases of American Cutaneous Leishmaniasis, mean population of the period, and mean incidence according to Pernambuco (PE) Health Region, for the first (2008-2012) and second quinquenium (2013-2017).

Health Region	Cases		Population		Incidence	
	Quinquenium 1	Quinquenium 2	Quinquenium 1	Quinquenium 2	Quinquenium 1	Quinquenium 2
I	157,2	87,8	3908757	4144599	4,0	2,1
II	43,4	28,2	566331	592135	7,7	4,8
III	133,8	94,4	574905	608681	23,3	15,5
IV	53,4	30,0	1241469	1337431	4,3	2,2
V	4,6	6,8	513660	537002	0,9	1,3
VI	1,0	0,6	382602	412820	0,3	0,1
VII	2,4	0,6	138719	145716	1,7	0,4
VIII	0,4	1,0	434713	483040	0,1	0,2
IX	7,2	6,4	327866	347718	2,2	1,8
X	0,4	0,4	180780	187847	0,2	0,2
XI	4,4	5,2	223879	235691	2,0	2,2
XII	25,2	35,6	302767	287792	8,3	11,4
PE	417	280	8796448	9320472	4,7	3,0

**TABLE 2:** Distribution of American cutaneous leishmaniasis cases according to sociodemographic and clinical epidemiological variables, Pernambuco, Brazil, 2008-2017.

Variable		Number of cases	%
**Age range**	<10 years	425	12,2
	10-19 years	739	21,2
	20-39 years	1.060	30,3
	40-59 years	802	23,0
	60-79 years	410	11,7
	80+ years	57	1,6
**Gender**	Female	1.390	39,8
	Male	2.102	60,2
**Race/color**	White	638	18,3
	Black	270	7,7
	Yellow	40	1,1
	Brown	2.198	62,9
	Indian	54	1,5
	Ignored/blank	293	8,4
**Schooling**	Illiterate	296	5,8
	Incomplete elementary school	1.630	46,7
	Complete elementary school	128	2,5
	Incomplete high school	83	1,6
	Complete high school	92	1,8
	Incomplete higher education	10	0,3
	Complete higher education	10	0,2
	Ignored/blank	966	18,9
	Not applicable	278	5,4
**Residence area**	Urban	854	24,4
	Rural	2.467	70,6
	Peri-urban	44	1,3
	Ignored/blank	128	3,7
**Clinical form**	Cutaneous	3.395	97,2
	Mucosa	98	2,8
**Epidemiological classification**	Autochthonous	2.754	78,8
	Allochthonous	118	3,4
	Undetermined	621	17,8
**Confirmation criterion**	Laboratory	1.029	29,5
	Clinical-epidemiological	2.464	70,5
**Case evolution**	High by cure	2.887	82,7
	Abandon	41	1,2
	Death by ACL	2	0,1
	Death by other causes	9	0,3
	Transference	10	0,3
	Ignored/blank	544	15,6

### Socioeconomic factors correlated with ACL

Infrastructure and poverty indices (Table 3) increased the estimates of the incidence of ACL. For every 0.01 increase in infrastructure, the incidence estimate increased by 2.6%. For every 0.01 increase in poverty, the estimated incidence of ACL increased by 6.7%. 

**TABLE 3:** Estimates of the zero-adjusted gamma regression model (ZAGA model) for correlation between ACL index in Pernambuco and socioeconomic variables.

Parameter	Coefficient	Estimate	p-value
µ	Intercept	-3,96	0,01
	Infra	2,58	0,01
	Poverty	6,47	0,00
σ	Intercept	0,02	0,78
ν	Intercept	2,69	0,00
	Infra	-2,92	0,00

### Spatial analysis

**FIGURE 1:**
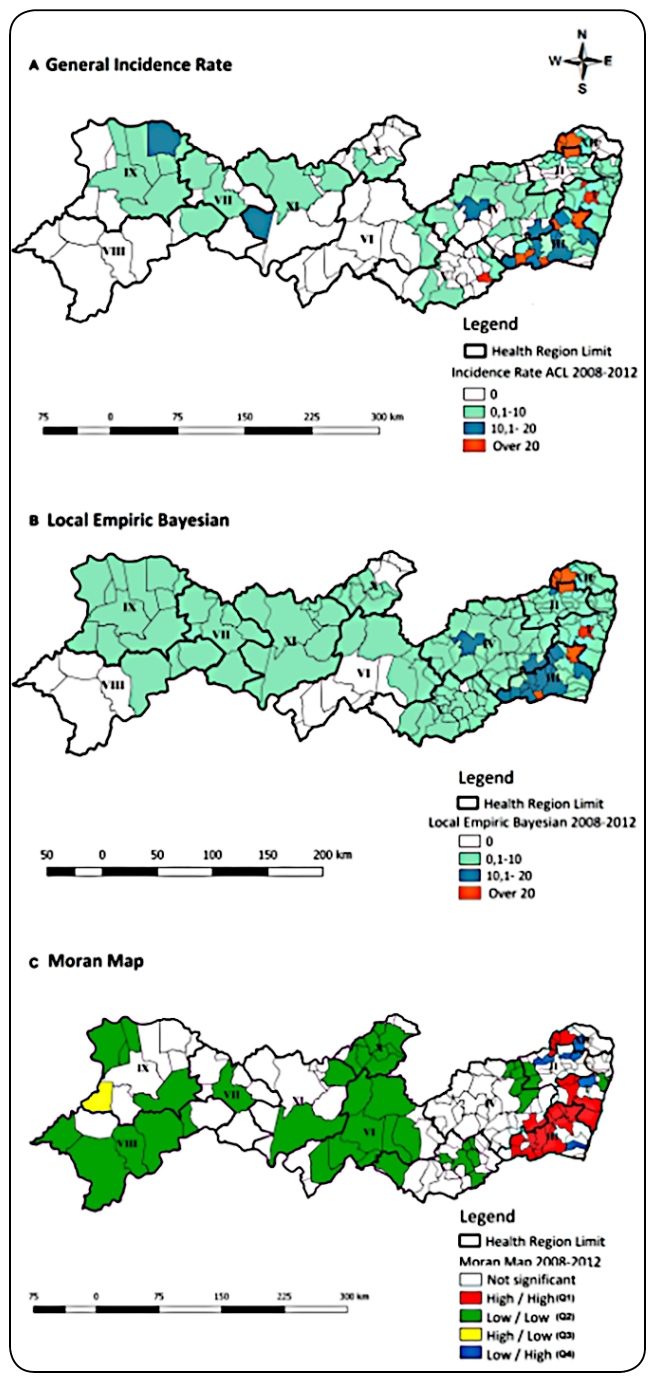
Spatial distribution of ACL gross incidence rate **(A)**, smoothed rate by Bayesian local empirical method **(B)**, and Moran Map **(C)** per 100,000 inhabitants, Pernambuco, Brazil, 2008-2012.

In 2008-2012 quinquennium, Pernambuco’s incidence was 4,9/100,000 inhabitants (0/100,000- 104.8/100,000 inhabitants). In 22 municipalities (11,9%) located in Health Regions I, II, III, IV, and XII, the incidence rate was more than 20,0/100,000 inhabitants(Figure 1A). The Bayesian analysis indicates a reduction of these to 17 (9.2%) (Figure 1B). In Moran Map, 23 municipalities with high intervention priority (Q1) were distributed in two clusters: one comprised 19 municipalities (Regions I, III, and IV), while the other comprised four municipalities (Regions II and XII). Intermediate priority areas were located in Health Regions I, II, III, and XII (Figure 1C).

**FIGURE 2:**
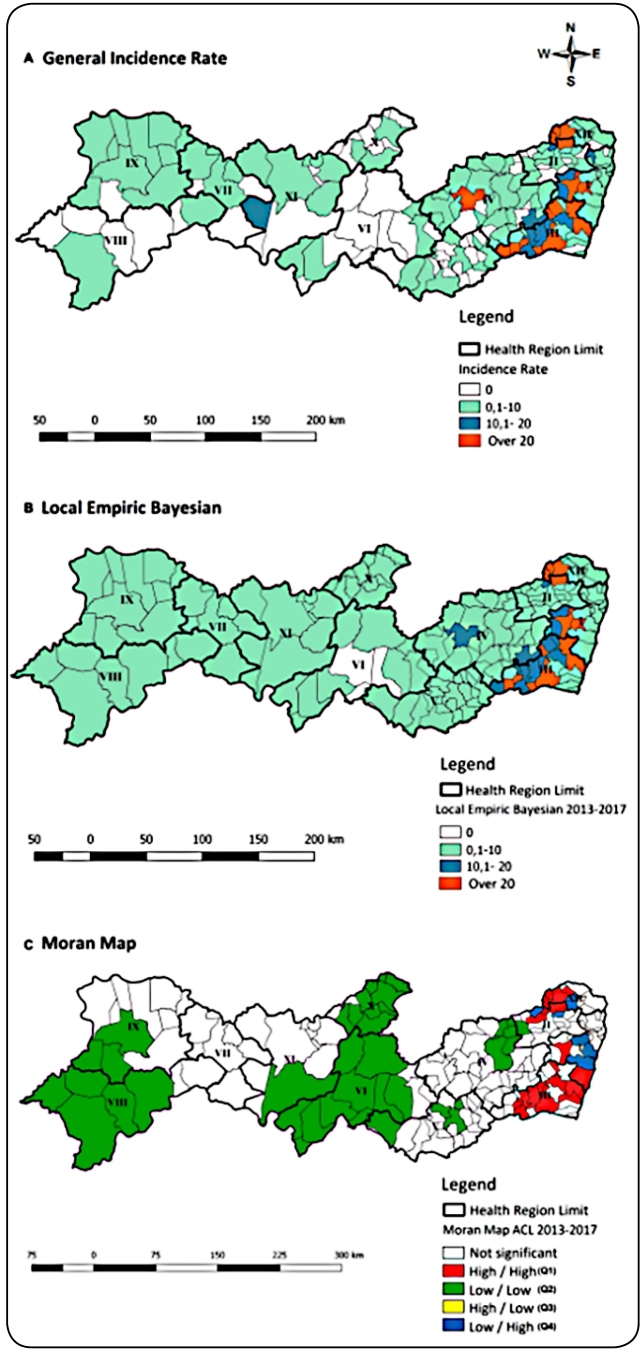
Spatial distribution of ACL gross incidence rate **(A)**, smoothed rate by Bayesian local empirical method **(B)**, and Moran Map **(C)** per 100,000 inhabitants, Pernambuco, Brazil, 2013-2017**.**

In 2013-2017 quinquennium, incidence was lower (3,2/100,000 inhabitants) than the first -from 0/100,000 to 95,8/100,000 inhabitants. In 13 (7,1%), incidence rates in Regions I, II, III, IV, and XII were more than 20,0/100,000 inhabitants (Figure 2A). After Bayesian smoothing, decreased to seven (3.8%) (Figure 2B). In Moran Map, 19 municipalities were identified as Q1, and they were divided into two clusters: the first comprised 12 municipalities in Regions I and III, while the other comprised 7 municipalities in Regions II and XII. The incidence decreased in the seven municipalities, while increased in three municipalities compared with the respective clusters in the previous period. In addition, six municipalities with intermediate priority were identified in the Regions I, II, and XII (Figure 2C).

## DISCUSSION

This study showed that the most individuals affected by ACL were younger adults, were men, had brown race/color, living in rural areas, and with low educational level. Majority of the individuals acquired the infection at the place of residence; they developed the cutaneous form, diagnostic on the clinical epidemiological criterion and evolved to cure. The disease was present in the large part of the state, but with heterogeneous incidence rates compared with municipalities. There were two high priority intervention clusters covering the municipalities in Health Regions I, II, III, IV and XII. 

When analyzing the total period and the first quinquennium, we noted a decrease in the incidence rates, which agrees with the national data^3^. However, there was an increase in the incidence rates in the second period. Such casuistry was observed in Brazil from 1985 onwards, where an increasing number of cases presented transmission peaks every 5 years^3^, corresponding to the increasing incidence observed in the provinces of Iran^29^ ^,^ ^31^ ^,^ ^32^ and Ethiopia^30^. Moreover, the increased incidence may be related to the anthropic actions due to socioeconomic pressure, especially the disordered urbanization that contributes to the domination of the sandfly responsible for transmission of the disease^10^ ^-^ ^13^.

Our analysis showed that ACL was more frequent in brown-skinned individuals, those aged 20 and 39 years, and men, which is similar to the profile observed in Brazil^33^ ^,^ ^34^ and Argentina^35^. Regarding the level of education, most of the patients were unable to complete primary education. This finding corroborates with those of previous studies, indicating the association between the occurrence of neglected infectious parasitic diseases and low-educated populations^18^ ^,^ ^34^ ^,^ ^35^, even implying the preventive practice of the disease^36^.

Regarding the residence area, ACL were more common in rural areas with autochthonous transmission. This result is consistent with the literature data, which indicated that the disease was a zoonotic disease; people in contact with forests and rural workers are more predisposed to acquiring ACL as they are more often exposed to the vector and reservoir^3^ ^,^ ^18^ ^,^ ^37^. This pattern has been progressively modified; the disease has been occurring in urban and peri-urban areas^13^ ^,^ ^38^ ^,^ ^39^ as a result of environmental modifications and demographic changes^12^ ^,^ ^14^ ^,^ ^40^.

The cutaneous form were predominant among patients, which is consistent with the results of other studies^5^ ^,^ ^41^. Choice’s confirmation criteria used to diagnose most of our study patients were epidemiological and clinical factors; the use of this criterion is based on three aspects-clinical suspicion, impossibility to access laboratory diagnosis methods, and the link with disease transmission areas^3^. That result reflects the need to expand access to complementary parasitological exams, which not only helps detect *Leishmania* stocks^42^ but also contributes to differential diagnosis^43^.

Evolution to a curative stage was the common outcome. Such evolution occurred spontaneously, which is the reason why the disease was classified as pseudobenign, thus preventing the appropriate diagnosis, treatment, and monitoring of affected patients^44^. Although death due to ACL is rare, the disease reflects a negative impact on patients’ daily lives. That may cause individuals to develop incapacitating lesions resulting in psychological, social, and economic issues; affecting individual’s interpersonal relationships and life quality^45^ ^,^ ^46^. According to Bezerra et al. (2016)^47^, in Brazil, the rate of disability due to the occurrence of lesions in 2016 corresponded to an average of 1-2 years of life lost per 100,000 inhabitants, which increases with age.

When we verified the correlation with socioeconomic factors, we found that infrastructure and poverty tend to increase the incidence of ACL. The first factor can be justified by the proliferation and urbanization of the vector^10^ ^-^ ^13^ ^,^ ^40^. The second factor presented a greater relative increase, which is congruent with the reports of previous studies conducted in Matara (Sri Lanka)^48^ and India^49^, where low income had a significant association with high incidence.

These findings can be confirmed when the priority areas of intervention are verified, since the Region III presented the highest incidence of ACL and, according to the Human Development Atlas of Brazil^50^, coincided with the areas with high rates of vulnerability to poverty. Health Regions I and IV also had high incidence of ACL; however, these areas had high per capita income^50^. This is possibly because there are socioeconomic differences between the municipalities in the same health region. This finding suggests that inequality is a strong risk factor^16^ ^,^ ^19^.

The water and sewage situation in many municipalities in Pernambuco is precarious. Regions II and III were the main areas that were not able to avail of the garbage collection service and had inadequate basic sanitation^50^. Valero and Uriarte (2020)^51^ stated that the lack of water supply, sewage system, and garbage collection are risk factors for ACL because poor environmental conditions are good breeding grounds of sandflies.

The spatial analysis revealed two priority clusters for intervention; the first group consisted of municipalities belonging to the Health Regions I, III, and IV, while the second group consisted of municipalities belonging to the Health Regions II and XII. Areas considered transitional were also identified predominantly in these regions. Historically, the region where the clusters were identified, Pernambuco’s Zona da Mata, had 60% of ACL cases in the state^46^ ^,^ ^52^ ^,^ ^53^ ^,^ ^54^. This region geographically corresponds to the area with high concentration of primitive Atlantic Forest remnants; in addition, it presents particularities that favor the disease’s spread such as tropical humid climate, rural activities^53^, and accentuated social inequality^55^. Other studies have identified areas that are most vulnerable to the spread of the disease, such as the Minas Gerais (BR), which were subdivided into three clusters (those with rural activities, forest areas, and unusable lands)^18^, and a rural municipality in Venezuela with five clusters (those with a strong influence of environmental factors). Thus, the characteristics of the areas become a focal character in the distribution of the disease^56^. The number of municipalities in the cluster located in Health Regions I, III, and IV were fewer than those in the previous quinquennium. The opposite of it occurred with the cluster of the Health Regions II and XII; this dynamic may be related to the prevention and control of activities as well as the sensitivity to effective notification in municipalities, respectively. It is necessary that such interventions act jointly to guarantee efficacy due to the high transmission potential and disease magnitude^3^. Primarily, it is necessary to identify the disease ecology and control the main reservoirs and vectors besides health education by participating in community programs; as a secondary prevention, early diagnosis and timely treatment should be observed^43^. It is up to the surveillance and healthcare team to improve the information systems application and to involve the healthcare professionals through permanent education.

Moreover, the social determinants of health established by the WHO (2011)^57^ regarding the impact on equity and well-being in the socioeconomic and political context and in the behavioral, biological and psychosocial context must be highlighted. The inequalities in these structures generate an environment conducive to the reproduction of neglected diseases, such as the ACL. Thus, to improve the health situation, it is necessary to reduce inequities; improve social performance, such as well-being, education, social cohesion and conservation of the environment; increase productivity; and improve economic development.

The data described in this study were extracted from SINAN, an important system for epidemiological information knowledge. Notwithstanding, secondary data should be carefully interpreted, as it may lead to inconsistencies during information processing and underreporting. To address the limitations, the duplicity and incompleteness of the cases were analyzed. Epidemiological bias may exist in the attempt to interpret the heterogeneity of the distribution of rates in the geographical space and the socioeconomic indicators analyzed.

Therefore, this study shows that combining spatial analysis tools and epidemiological indicators optimizes the exploitation of the ACL temporal space, helping to locate risk areas, and subsidizing the planning of prevention and control actions in the priority and intermediate areas of intervention.

The disease has been persistent in the Pernambuco’s Zona da Mata, which is in the Health Regions I, II, III, and XII; hence, it is important to establish and strengthen the indissociable measures of preventive, diagnostic, and control against the disease in this region. Moreover, the incidence rates in certain areas should be monitored. A few studies reported the association between ACL distribution and socioeconomic risk factors; however, further investigations should be conducted to determine the other risk factors of this neglected disease and thus establish the appropriate health and social policies, aimed improved the disease control.
